# Supplementary figures and images for: The effect of cooperator recognition on competition among clones in spatially structured microbial communities
Source: PLoS One. 2024 Mar 28;19(3):e0299546. doi: 10.1371/journal.pone.0299546 (PMC10977701; doi:10.1371/journal.pone.0299546)

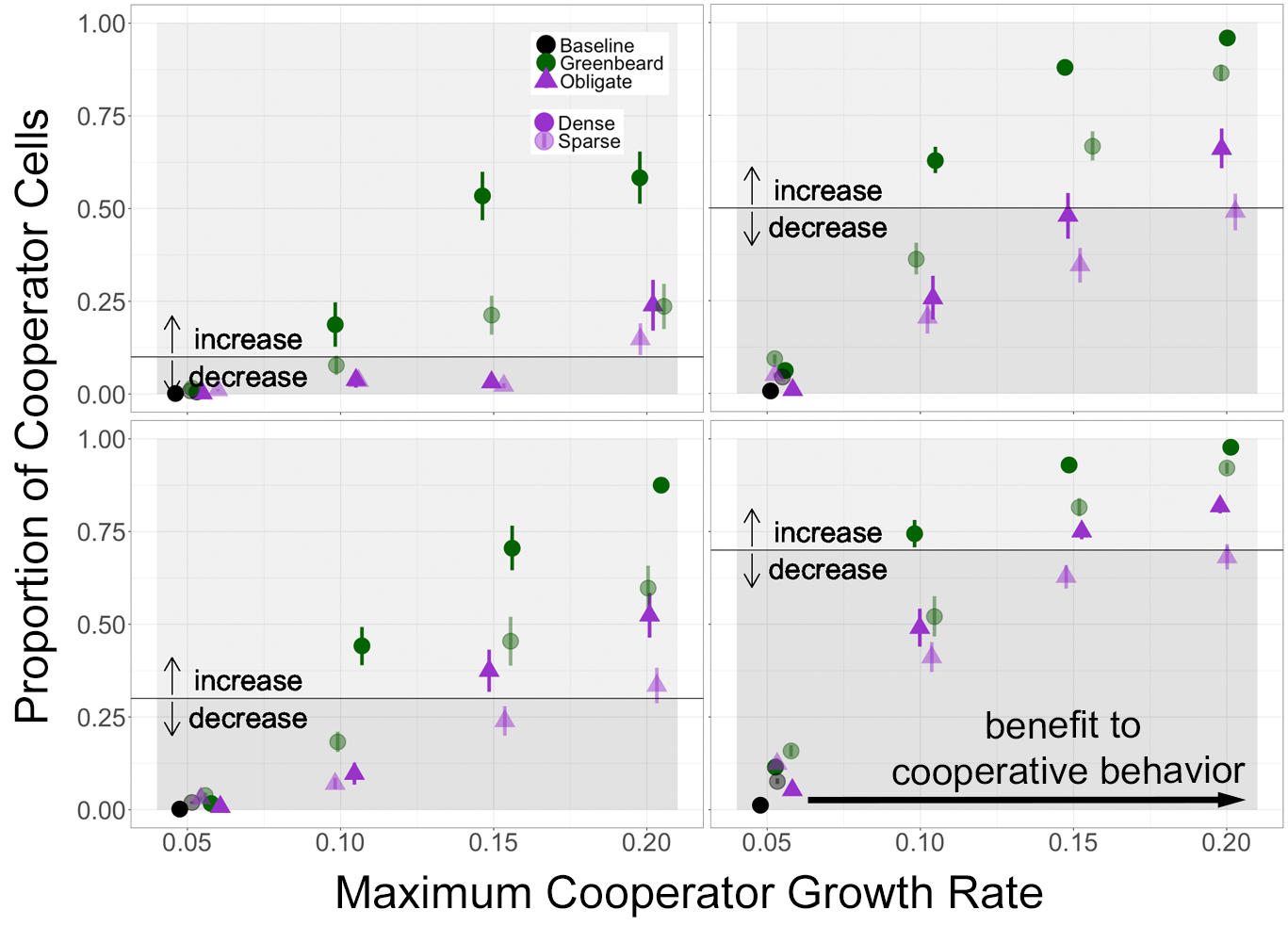

Supplement: S1 Fig — Total proportion of slow strain (S) when colonies are sparse (light) or dense (solid). Strain S are cooperators and strain F are non-cooperators; purple triangles represent obligate cooperation and green circles represent greenbeard cooperation. The horizontal axis is the maximum growth rate for strain S. Each panel represents a different initial proportion of S. Error bars are standard error of the mean. (TIF) [file pone.0299546.s001.tif]
