# Supplementary material for: The effect of cooperator recognition on competition among clones in spatially structured microbial communities
Source: PLoS One. 2024 Mar 28;19(3):e0299546. doi: 10.1371/journal.pone.0299546 (PMC10977701; doi:10.1371/journal.pone.0299546)
Supplement: S1 File — Description of growth, competition, and cooperation in the simulations. (PDF) [file pone.0299546.s002.pdf]

Supporting Information

A. Simulation Method

To model cell growth occurring in continuous time, the simulation used a discrete time Monte Carlo method with a small time step $\Delta t = 0.1$ and random sequential updating. A cell was selected at random for possible division. Its growth rate r (either r_S or r_F depending on cell type) was calculated from Eq. (1) or (2), and its probability to grow was $P = r\Delta t$. A uniform random number $0 \leq u < 1$ was generated, and if $u < P$, the cell divided and a daughter cell was placed nearby. For each time step, a random cell selection was made T times, where T was the total number of cells in the colony at the beginning of the time step.

Initial condition

We began the simulation with a diluted inoculum of cells into a small square at the center of the bottom layer of our cubic array (see Fig. 1). The size of the cubic array was $N_c = 50$ cells (x-direction) by 50 cells (y-direction) with a height of $N_z = 100$ cells (z-direction). The initial square had dimensions $d \times d$, where in our simulations $d = 15$, and a fraction $f = 0.05$ of the sites were randomly selected to fill with cells (rounded down if $f\,d^2$ is not an integer). As random sites were selected, slow cells were placed until the allotted fraction f_S of slow cells was reached, after which the remaining initial cells were the fast type.

Local densities

To determine the effect of social interactions on a cell, we must calculate the local density of each cell type near the cell of interest. The density of slow cells, ϕ_S, was the number of slow cells in a cube of volume $(2R_i + 1)^3$ centered at the focal cell, divided by the volume $(2R_i + 1)^3$. Here, R_i is the interaction radius. The local density of fast cells, ϕ_F, was defined similarly. If a cell was near the boundary of the cubic array, we divided by the actual volume that surrounded the cell and was part of the array. The local densities were between 0 and 1.

Calculating the local densities could be computationally expensive because of the need to loop over many nearby cells, so we reduced how often they were calculated. At the beginning of the simulation, local densities were computed for each cell. They were recomputed only when a cell was being considered for division and its neighborhood had been altered. We used a flag for

each cell to track whether its local densities were current or its neighborhood had potentially been altered by movement of nearby cells.

Division: Finding an empty site and cell movement

If it was determined that a cell would divide, the next step was to find an empty site for the daughter cell to be placed. Similar to Momeni et al. (2013), we first checked the eight sites immediately surrounding the selected cell in the same horizontal plane, giving priority to the four sites directly adjacent rather than diagonally adjacent to the cell (see Figure 1a). If more than one equidistant empty site was available, we randomly selected one of those empty sites. The daughter cell was placed in this empty site, adjacent to the parent, and no cells moved.

If the surrounding eight sites were full, we next considered whether the dividing cell would push nearby cells aside horizontally in a planar displacement neighborhood to make room for the daughter cell. We checked for empty sites within a square neighborhood of radius R_d in the same horizontal plane (see Figure 1b). Keeping track of all the empty sites that were found, we calculated the Euclidean distance to each site center. In other words, we used array coordinates to identify cell center. We found the minimum distance, and if there were multiple locations at the minimum distance, we selected between them at random. The daughter cell was placed adjacent to the parent, pushing the existing cells toward the empty site.

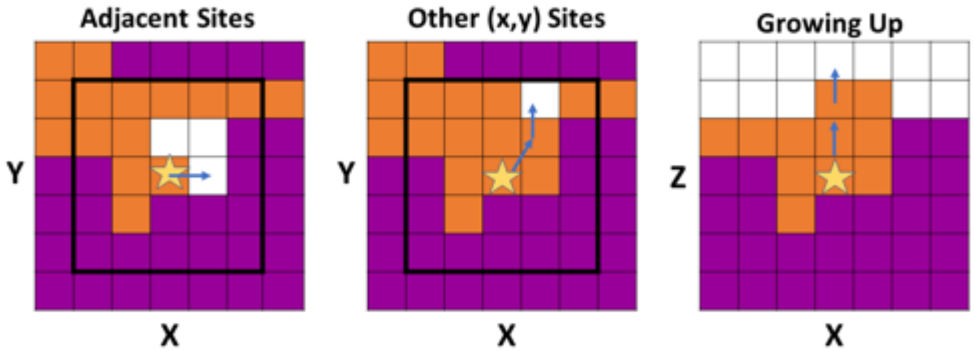

Figure 1 (a) A dividing cell (star) places daughter cell into a horizontally adjacent empty site. (b) A dividing cell finds the optimal path and accordingly pushes the cells on the path to the empty site, to create space to place its daughter. (c) A dividing cell places daughter directly upwards. The bold black square in (a) and (b) is the displacement neighborhood. In these figures, R_d = 2. This figure is adapted from Momeni et al. (2013), figure 1-S1.

The goal was to push the cells in a straight line toward the empty site, but the discreteness of the sites prevented doing this. We determined the optimal path along which to push cells as follows. From the dividing cell, we considered three adjacent cells: those in the x and y directions and the diagonally adjacent cell. Creating a vector from the dividing cell to each of those three cells, we determined which vector was most parallel to the vector from the dividing cell to the empty cell (see Figure 2). While the algorithm defined in Momeni et al. (2013) focused on distance and angle, our algorithm focused on making sure we remained as parallel as possible to the shortest path to the chosen empty site. To do this, we found the cosine of the angle between each vector and the main vector to the empty site. The cosine of each angle was calculated using the magnitudes and dot products of each of the three vectors with the main vector. The vector that created the smallest angle, or the largest cosine value, was the direction we selected to move closer to the empty site. If there was more than one direction with the maximum cosine value, we randomly chose between them. Having selected the first cell to be pushed by the daughter cell, we recorded its location. Next, we repeated the process to find the destination of the first cell being pushed toward the empty site. We continued finding cells to be pushed in the selected direction until we reached the empty site. The daughter cell was then placed next to the parent cell, and the displaced cells were pushed toward the empty site along the path that we found.

If no empty sites were found in the same plane within the displacement radius, we moved up in the z-direction (Figure 1c). Any cells above the dividing cell were pushed upward.

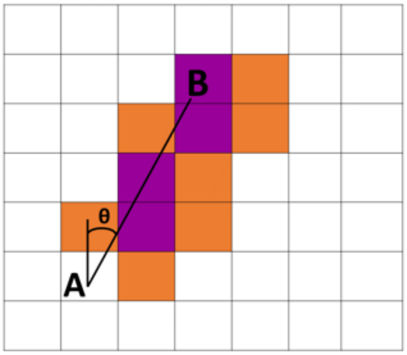

Figure 2 Finding the optimal path from a dividing cell at site A to an empty site B. The optimal path is along the purple sites, and the orange sites are the other neighboring sites considered.

B. Total Proportion of Slow Cells in Various Colony Densities

Total proportion of slow cells is plotted in Figure S1. For a denser setting, we used the measurement of total proportion of slow cells at the end of the simulation, when cell totals reached 50,000 cells. This was analogous to a natural setting and is shown by the solid purple circles. We varied the maximum growth rate for slow cells as well as the initial fraction of slow cells in the inoculation droplet.

References

Momeni, B., Brileya, K.A., Fields, M.W., Shou, W., 2013. Strong inter-population cooperation leads to partner intermixing in microbial communities. eLife 2, e00230. https://doi.org/10.7554/eLife.00230.001
