# Supplementary material for: The effect of cooperator recognition on competition among clones in spatially structured microbial communities
Source: PLoS One. 2024 Mar 28;19(3):e0299546. doi: 10.1371/journal.pone.0299546 (PMC10977701; doi:10.1371/journal.pone.0299546)
Supplement: Attachment — Submitted filename: Response To Reviewers.pdf [file pone.0299546.s005.pdf]

WILLIAM & MARY
CHARTERED 1693

Helen Murphy
Class of 1955 Distinguished Associate Professor
Department of Biology
P.O. Box 8795 / Williamsburg, VA 23187-8795
(757)-221-2216 / hamurphy@wm.edu

November 27, 2023

Dear Editor and Reviewers,

We thank you for the constructive and useful feedback on our manuscript, and for the effort you put into reviewing it. We believe our manuscript is stronger thanks to this review process.

Sincerely,

Helen Murphy (on behalf of all authors)

Editor's Summary:

Two reviewers have reviewed your original submission. Both reviewers indicated a minor revision was required. Reviewer one would like more to see more information on the modeling methods (and a minor fix to a figure legend) while reviewer 2 would like you to address how the simulations connect with natural systems. To me, the latter could be done in the discussion and I believe that this is what the reviewer is alluding to. I look forward to reading your revised manuscript.

Reviewer #1:

(1) The manuscript titled "The effect of cooperator recognition on competition among clones in spatially structured microbial communities" describes an agent based modeling approach where cooperation in biofilm is assessed. An important feature is that the models incorporate kin recognition/selection and by doing so the can model the effects of it in spatially structured environments. The tested conditions are biologically relevant.
The paper is clearly written and the results make intuitive sense. The authors are clear about the limitations of the study.

As the models are not shared and I am not an expert on these type of models I can not assess those details.

While the general concepts are well described in the methods section it is not mentioned what software was used for the modeling and I think the models itself should be made available as well.

We have added a line in the Methods that notes the simulations were written in Python and have made the code available through GitHub. We have done our best to comment extensively in the code.

(2) Figs 1,2,5: From the legend it is not clear what is on the x,y and z axis of the 3D plot

The figures with colonies represent the simulations. The axes provide the location for the cells in the simulated cubic array and are in units of cell length. We have updated the text to clarify this in Figures 1

and 2.

(3) The legend of Fig 6 needs to be extended. In the current form I do not understand what the authors are trying to say with Figure 6a for instance and how it links to the other panels.

Figure 6a is showing the different strategies being competed against each other and should be used as a key for interpreting Figure 6b. We have updated the legend to clarify.

Reviewer #2:

This study investigated the effects of different types of cooperation in a microbial colony using spatially explicit, agent-based simulations of two interacting strains. They found scenarios that favor a form of kin recognition in spatially structured microbial communities. The observed results suggest that in some ecological scenarios, recognition mechanisms may be beneficial even in spatially structured communities. This study is interesting and well organized. My major concern is on how to build relationships between this simulation results to ecological systems. As we know the environment selects the microorganisms. So how to determine the different roles of environmental conditions and microbial interactions on microbial community development. Please discuss these contents in the manuscript. I think it could be accepted by minor revisions.

We appreciate the encouragement to connect our simulation and its results to the actual biology of microorganisms. It is absolutely true that the environment selects the organism. However, when multiple lineages of a single species find themselves together in an environment conducive to growth, they will be interacting with each other. We are not sure we can answer the question of how to determine the role of the environment vs. social interactions in structuring microbial communities, but we have updated the end of the discussion to provide plausible biological scenarios in which multiple lineages of one species could find themselves growing and interacting.
